# Mechanism of oxidant-induced mistranslation by threonyl-tRNA synthetase

**DOI:** 10.1093/nar/gku271

**Published:** 2014-04-15

**Authors:** Jiang Wu, Yongqiang Fan, Jiqiang Ling

**Affiliations:** 1 Department of Microbiology and Molecular Genetics, Medical School, University of Texas Health Science Center, Houston, TX 77030, USA; 2 Graduate School of Biomedical Sciences, University of Texas, Houston, TX 77030, USA

## Abstract

Aminoacyl-tRNA synthetases maintain the fidelity during protein synthesis by selective activation of cognate amino acids at the aminoacylation site and hydrolysis of misformed aminoacyl-tRNAs at the editing site. Threonyl-tRNA synthetase (ThrRS) misactivates serine and utilizes an editing site cysteine (C182 in *Escherichia coli*) to hydrolyze Ser-tRNA^Thr^. Hydrogen peroxide oxidizes C182, leading to Ser-tRNA^Thr^ production and mistranslation of threonine codons as serine. The mechanism of C182 oxidation remains unclear. Here we used a chemical probe to demonstrate that C182 was oxidized to sulfenic acid by air, hydrogen peroxide and hypochlorite. Aminoacylation experiments *in vitro* showed that air oxidation increased the Ser-tRNA^Thr^ level in the presence of elongation factor Tu. C182 forms a putative metal binding site with three conserved histidine residues (H73, H77 and H186). We showed that H73 and H186, but not H77, were critical for activating C182 for oxidation. Addition of zinc or nickel ions inhibited C182 oxidation by hydrogen peroxide. These results led us to propose a model for C182 oxidation, which could serve as a paradigm for the poorly understood activation mechanisms of protein cysteine residues. Our work also suggests that bacteria may use ThrRS editing to sense the oxidant levels in the environment.

## INTRODUCTION

Aminoacyl-transfer ribonucleic acid (tRNA) synthetases (aaRSs) are essential enzymes that ligate cognate amino acids to tRNAs, thereby providing the ribosome with correct aminoacyl-tRNAs (aa-tRNAs) as building blocks for protein synthesis (1). Due to the structural similarity, some near-cognate amino acids are not sufficiently discriminated at the aminoacylation site. To ensure translational fidelity, aaRSs use pre- and post-transfer editing mechanisms to hydrolyze misactivated amino acids and misacylated aa-tRNAs, respectively (2,3). Whereas pre-transfer editing is often catalyzed by the aminoacylation site (4–6), post-transfer editing occurs at a distinct editing site that is either appended to the aaRS (7–12) or present as a free-standing protein (13,14). Impairing post-transfer editing activity causes increased translational errors (mistranslation) in bacterial and eukaryotic cells (10,15,16).

Severe mistranslation leads to growth inhibition in bacteria (16,17), mitochondrial dysfunction in yeast (18) and apoptosis in mammalian cells (19). It has also been shown that mice with compromised editing function in alanyl-tRNA synthetase (AlaRS) develop neurodegenerative symptoms (20). On the other hand, various levels of mistranslation appear to be tolerated by different types of cells (18,21), and mistranslation may even be advantageous under certain stress conditions (22–24). Translational errors can be increased by mutations (25,26), aminoglycoside antibiotics (27), nutrient starvation (28), viral infection (23) and oxidative stress (23,29). Our previous studies show that hydrogen peroxide (H_2_O_2_) impairs the editing function of *Escherichia coli* threonyl-tRNA synthetase (ThrRS) and causes serine (Ser) misincorporation at threonine (Thr) codons (29). The editing site of bacterial and eukaryotic ThrRSs contains a conserved cysteine residue (C182 in *E. coli* ThrRS) that is critical for the hydrolysis of misacylated Ser-tRNA^Thr^ (30,31). We have shown that C182 is the target for H_2_O_2_-mediated oxidation, but how C182 is activated for oxidation remains unclear (29).

Cysteine oxidation represents a wide-spread, yet under-characterized group of post-translational modifications and plays important roles in the cell (32). In particular, cysteine sulfenic acid (RSOH) results from reaction of protein cysteines with various oxidants and can be reversed to cysteines by reducing agents (33). Sulfenylation alters the structural and functional properties of cysteine and has been found in an increasing number of proteins, many of which are involved in redox regulation and cell signaling (34,35). The activation mechanisms of protein cysteines are not well-understood, making it difficult to predict reactive cysteines based on sequence and structural information. In this work, we demonstrate that C182 of *E. coli* ThrRS is modified to cysteine sulfenic acid by air, H_2_O_2_ and hypochlorite, and clarify the mechanism by which C182 is oxidized to regulate protein synthesis fidelity.

## MATERIALS AND METHODS

### Cloning, mutagenesis and general methods

*E. coli* ThrRS variants were cloned into pET28a vector (Novagen) with an N-terminal His tag. Expression of recombinant proteins was induced at 37°C for 4 h with 0.5 mM isopropyl β-D-1-thiogalactopyranoside in *E. coli* strain Rosetta pLysS in Luria-Bertani (LB) media. Wild-type (WT) ThrRS and ThrRS C812A were purified according to standard procedures using Ni-NTA resin (Qiagen). H73A, H77A and H186A mutations were generated using the QuikChange mutagenesis method.

### DAz-2 labeling of sulfenic acid *in vitro* in recombinant *E. coli* ThrRS

The method used to label RSOH in ThrRS was modified from previous publications (36,37). Briefly, purified WT and mutant ThrRSs were first treated with 10 mM dithiothreitol (DTT) at room temperature for 20 min to reduce any pre-existing reversibly oxidized forms of thiol group, including disulfide bonds and RSOH. After removing DTT with Bio-Spin 6 desalting columns (Bio-Rad), 5 μM or 2 μM protein was incubated with oxidants at different molar ratios and 40-fold excess of DAz-2 (Cayman Chemical) at 37°C for 1 h. In the experiments testing the effects of metals on RSOH formation, protein samples were incubated with 5-mM ethylenediaminetetraacetic acid (EDTA) on ice for 30 min. EDTA was then removed with Bio-Spin 6 columns before DAz-2 labeling in the presence and absence of metals.

### Biotinylation of DAz-2-labeled *E. coli* ThrRS and western blot analysis

DAz-2-labeled ThrRS was conjugated to biotin via Staudinger ligation with 0.25 mM phosphine-PEG3-biotin (Thermo Scientific) at 37°C for 2 h. Biotinylation reactions were terminated by the addition of 1 ml of cold acetone and were kept in −80°C for 20 min. The precipitated protein was centrifuged at 17 000 x *g* for 20 min. Protein pellet was washed once by 200 μl acetone then resuspended in 2x sodium dodecyl sulphate-polyacrylamide gel electrophoresis (SDS-PAGE) loading dye.

To detect the RSOH by western blot, biotinylated proteins were separated by 10% SDS-PAGE and transferred to PVDF membrane (Bio-Rad) with Trans-Blot SD Semi-Dry Electrophoretic Transfer Cell (Bio-Rad). The membrane was blocked in 3% bovine serum albumin (Fisher) in Tris-buffered saline Tween-20 (TBST; 25 mM Tris/Tris-HCl, 137 mM NaCl, 2.7 mM KCl and 0.05% Tween-20) at room temperature for 1 h. Blocked membrane was then incubated with 1:50 000 dilution of streptavidin-horseradish peroxidase (HRP) (GE Healthcare) at room temperature for 1 h, washed three times with 50 ml of TBST and developed with Clarity Western enhanced chemiluminescence (ECL) Substrate (Bio-Rad). Protein loading amount was confirmed by either HisProbe-HRP (Thermo Scientific) western blot or Ponceau S staining.

### tRNA assays *in vitro*

tRNA^Thr^ transcript was obtained using the T7 RNA polymerase runoff procedure *in vitro* as described (38). Aminoacylation experiments were performed at 37°C as described (10) in the presence of 100 mM Na-HEPES pH 7.2, 30 mM KCl, 10 mM MgCl_2_, 2 mM adenosine triphosphate (ATP), 25 μM [^3^H] Ser (25 μCi/ml or 275 cpm/pmole), 5 μM tRNA transcript or 4 mg/ml total *E. coli* tRNA and 5 μM ThrRS. EF-Tu was activated as described (39) in 50 mM Na-HEPES pH 7.2, 1 mM DTT, 68 mM KCl, 6.7 mM MgCl_2_, 2.5 mM phosphoenolpyruvate, 0.5 mM GTP and 30 μg/ml pyruvate kinase at 37°C for 20 min before use.

### Growth assay

The growth media contained 48 mM Na_2_HPO_4_, 23 mM NH4Cl, 22 mM KH_2_PO_4_, 8.5 mM NaCl, 2 mM MgSO_4_, 0.1 mM CaCl_2_, 1% glucose, 0.1 mM thiamine, 20 μg/ml each of the 20 proteinogenic amino acid except Ala, Cys, Thr and Ser, 0.02 U/ml *Aspergillus niger* glucose oxidase and 0.05 U/ml human myeloperoxidase. *E. coli* was grown in LB media overnight, washed twice with minimal media, diluted to A600 = 0.02 and grown at 37°C in the above media.

## RESULTS

### Editing site C182 in *E. coli* ThrRS is modified to sulfenic acid by multiple oxidants

**Figure 1.**
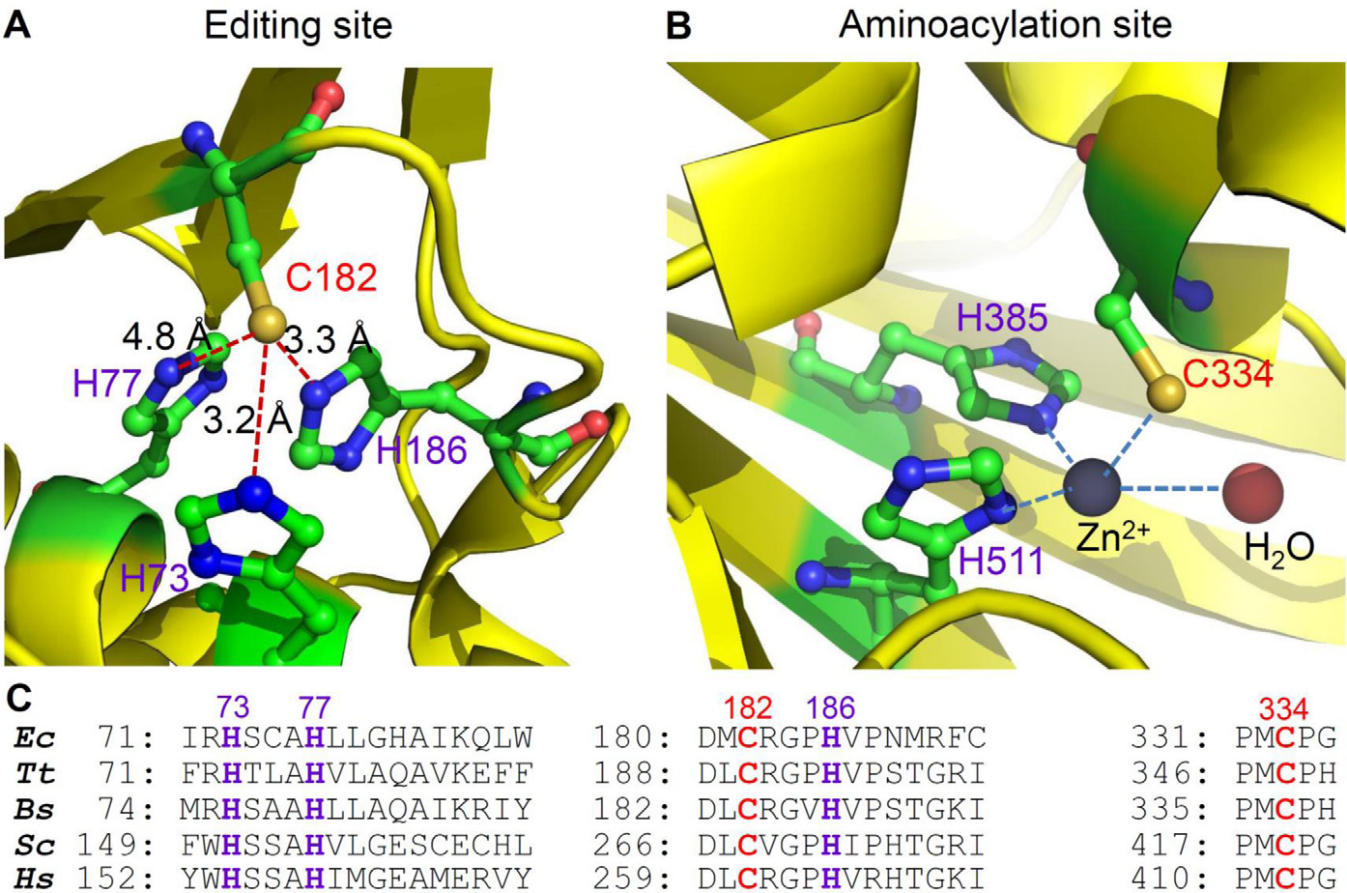
Editing and aminoacylation sites of *E. coli* ThrRS. (**A**) Crystal structure of ThrRS editing site (PDB: 1TJE). C182 is close to H73, H77 and H186. All four residues are critical for post-transfer editing of Ser-tRNA^Thr^ to maintain aminoacylation fidelity (31). The imidazole ring of H73 was re-annotated from the original structure (30). (**B**) Crystal structure of ThrRS aminoacylation site (PDB: 1EVK). C334, H385 and H511 coordinate a zinc ion, which is essential for aminoacylation. (**C**) Sequence alignment of conserved motifs in ThrRS editing and aminoacylation sites. *Ec*: *Escherichia coli*; *Tt*: *Thermus thermophilus*; *Bs*: *Bacillus subtilis*; *Sc*: *Saccharomyces cerevisiae*; *Hs*: *Homo sapiens*.

Bacterial and eukaryotic ThrRSs contain a conserved cysteine in the N-terminal editing site (9,30) (Figure 1). Structural and enzymatic studies of *E. coli* ThrRS suggest that the thiol group of C182 directly participates in the hydrolysis of Ser-tRNA^Thr^ through interaction with the 2′-hydroxyl group of the tRNA terminal adenosine (30,31). C182A and C182S mutations lead to loss of editing activity and Ser misacylation to tRNA^Thr^ (31). Our studies *in vitro* and *in vivo* show that oxidation of ThrRS by H_2_O_2_ causes editing defects and Ser misincorporation at Thr codons (29). Reducing the oxidized ThrRS with DTT or sodium arsenite (NaAsO_2_) recovers the editing activity, suggesting that a cysteine residue is reversibly oxidized (29).

**Figure 2.**
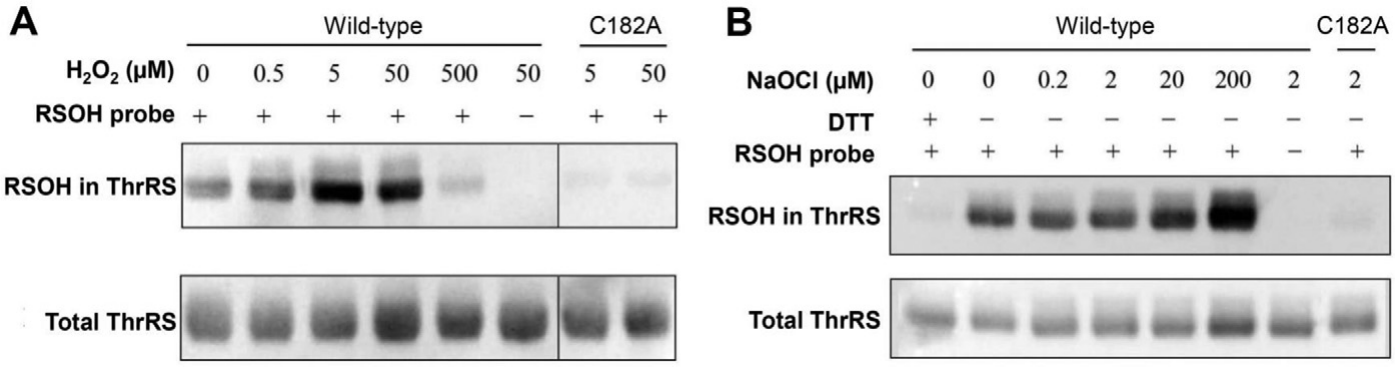
Sulfenic acid formation in *E. coli* ThrRS. Purified His_6_-tagged *E. coli* ThrRS wild-type (WT) and C182A variants [5 μM in (**A**) and 2 μM in (**B**)] were incubated with oxidants and DAz-2 (a chemical probe specific for RSOH) for 1 h. Labeled proteins were biotinylated and detected with streptavidin-conjugated HRP, and total ThrRS was revealed with a His probe. RSOH was detected in air-oxidized WT ThrRS, but not in the C182A mutant, suggesting that C182 is the target for oxidation. The RSOH level increased in the presence of H_2_O_2_ or NaOCl. A decrease of DAz-2 labeling at 500 μM H_2_O_2_ was likely due to over-oxidization of C182 to sulfinic or sulfonic acid. This experiment was repeated with representative results shown.

To test the oxidation product in ThrRS, we utilized a chemical probe that specifically labels sulfenic acid—4-(3-azidopropyl)cyclohexane-1,3-dione or DAz-2 (40). Purified WT ThrRS could be labeled with DAz-2 when the reducing agent (DTT) was removed from the protein buffer with a desalting column (Figure 2A and B). Adding DTT in the reaction abolished DAz-2 labeling (first lane, Figure 2B), suggesting that air oxidizes ThrRS to form RSOH. Treating WT ThrRS with 1:1 molar ratio (5 μM) of H_2_O_2_ or 20 μM sodium hypochlorite (NaOCl) substantially increased RSOH formation. In contrast, DAz-2 was unable to detect RSOH formation in the C182A mutant of ThrRS treated with H_2_O_2_ or NaOCl (Figure 2), supporting the notion that C182 is the sensitive target for oxidation in ThrRS.

### ThrRS oxidation increases misacylation of Ser to tRNA^Thr^

**Figure 3.**
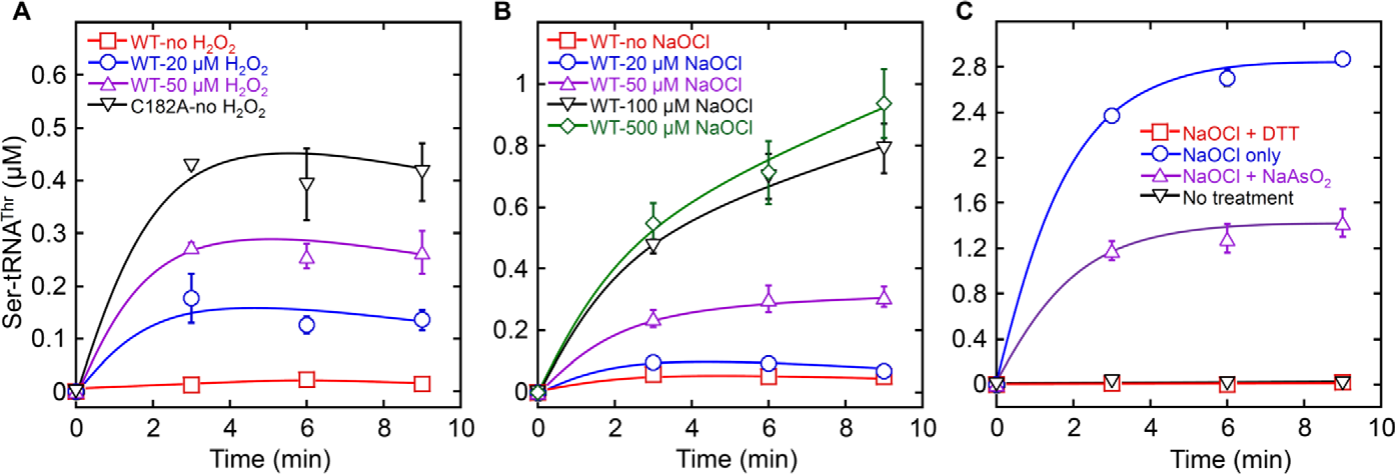
Serylation by oxidized *E. coli* ThrRS. (**A**) Five micromolar ThrRS and 25 μM [^3^H] L-serine were incubated at 37°C for 1 h in the presence and absence of H_2_O_2_. ATP (2 mM) and total *E. coli* tRNA (4 mg/ml) were added last to start the reaction. C182A was an editing-defective ThrRS mutant used as a control. (**B**) Five micromolar ThrRS and 25-μM [^3^H] L-serine were incubated at 37°C for 5 min in the presence and absence of NaOCl. ATP (2 mM) and total *E. coli* tRNA (4 mg/ml) were added last to start the reaction. (**C**) Five micromolar ThrRS and 25 μM [^3^H] L-serine were treated with 100 μM NaOCl for 5 min at 37°C, followed by treatment with DTT (11 mM) or NaAsO_2_ (11 mM) for 5 min. ATP (2 mM) and *E. coli* tRNA^Thr^ transcript (5 μM) were added last to start the reaction. The above results were the average of at least three repeats with error bars representing standard deviations.

Next, we tested how various oxidants affected the aminoacylation fidelity of ThrRS. Initial aminoacylation experiments performed *in vitro* showed that air-oxidized ThrRS did not accumulate Ser-tRNA^Thr^ in the reaction (Figure 3). Addition of 20 μM H_2_O_2_ or 50 μM NaOCl significantly increased the level of Ser-tRNA^Thr^. These results suggest that a threshold fraction of ThrRS editing sites need to be oxidized to cause misacylation in the absence of other translational factors. Subsequent treatment of NaOCl-oxidized ThrRS with DTT or NaAsO_2_ either abolished or decreased Ser-tRNA^Thr^ synthesis (Figure 3C), further supporting the formation of RSOH in the presence of NaOCl.

### Elongation factor Tu stabilizes misacylated Ser-tRNA^Thr^

**Figure 4.**
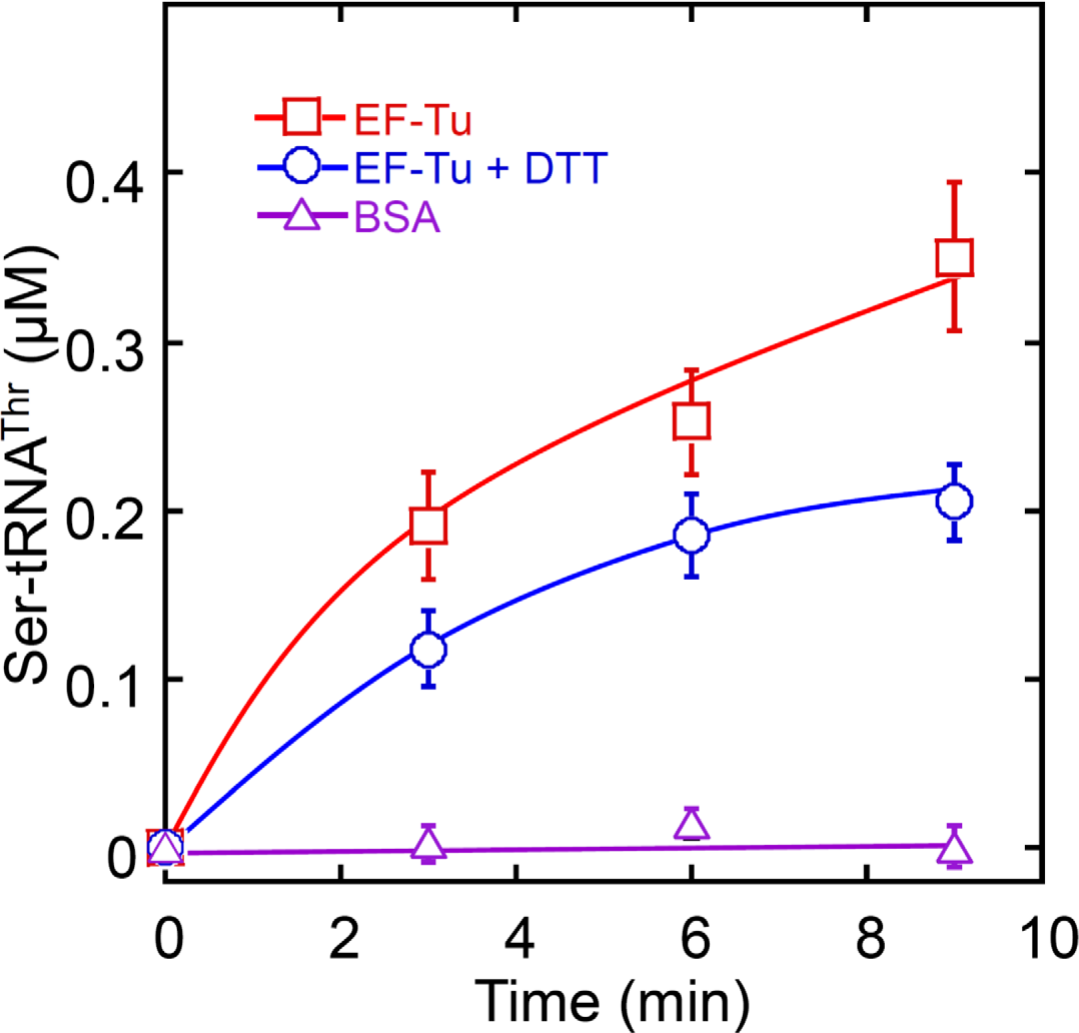
Serylation by *E. coli* ThrRS in the presence of EF-Tu. Five micromolar ThrRS and 25 μM [^3^H] L-serine were incubated at 37°C for 1 h in the presence and absence of DTT (10 mM). Activated EF-Tu (10 μM) was then added. ATP (2 mM) and total *E. coli* tRNA (4 mg/ml) were added last to start the reaction. In the absence of DTT, ThrRS editing site was partially oxidized by air (Figure 2), which increased Ser-tRNA^Thr^ formation. These results were the average of at least three repeats with error bars representing standard deviations.

Within the cell, aa-tRNAs are delivered to the ribosome by elongation factors (EF-Tu in bacteria and EF-1A in archaea and eukaryotes) (1,25). A previous kinetic competition model suggests that misacylated Tyr-tRNA^Phe^ dissociates from phenylalanyl-tRNA synthetase (PheRS); the editing site of PheRS then competes with EF-Tu to bind and hydrolyze Tyr-tRNA^Phe^ (39). A fraction of misacylated Tyr-tRNA^Phe^ may escape editing and become protected by EF-Tu. ThrRS and PheRS both belong to the Class II aaRSs (1), and it has been suggested that dissociation of aa-tRNA in Class II aaRSs is not the rate-limiting step during aminoacylation (41). To test the possibility that EF-Tu may compete with the ThrRS editing site for Ser-tRNA^Thr^, we performed serylation experiments in the presence of 5 μM ThrRS and 10-μM EF-Tu (Figure 4). Ser-tRNA^Thr^ formation was observed even in the presence of DTT, suggesting that EF-Tu competed with the reduced ThrRS editing site to partially stabilize the dissociated Ser-tRNA^Thr^. In the absence of DTT, air oxidation of ThrRS further increased Ser-tRNA^Thr^ formation, likely due to a decrease in editing efficiency. These data suggest that air oxidation of ThrRS may enhance the misacylation level in the cellular context.

### H73 and H186 are critical for oxidation of C182

**Figure 5.**
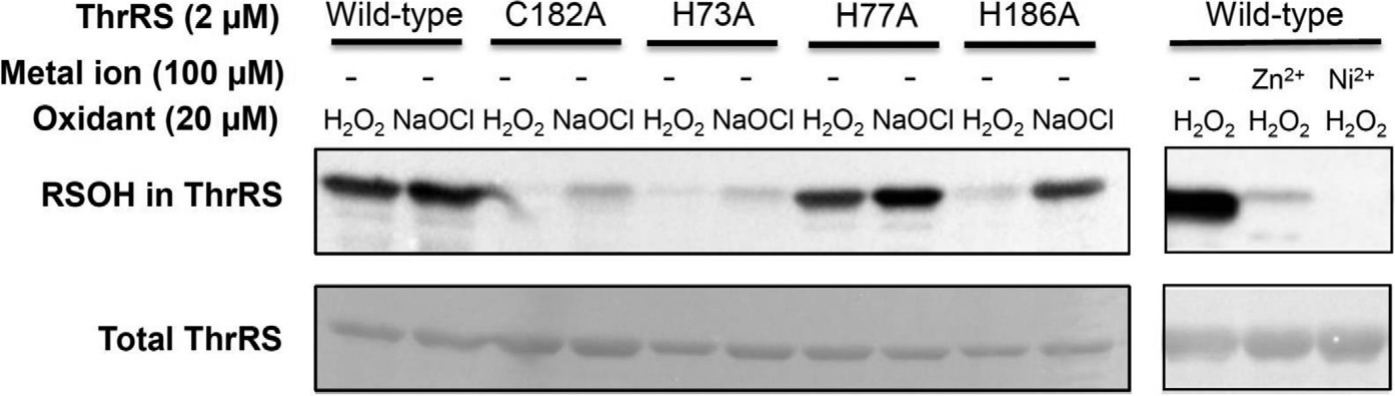
Sulfenic acid formation in ThrRS variants with and without metal ions. EDTA-treated ThrRS variants were incubated with oxidants and DAz-2 for 1 h in the presence and absence of metal ions. DAz-2 labeled proteins were biotinylated and detected with streptavidin-conjugated HRP, and total ThrRS was revealed with Ponceau staining. Mutating H73 or H186, but not H77, decreased RSOH formation at C182. Addition of ZnCl_2_ or NiCl_2_ inhibits ThrRS oxidation. This experiment was repeated with representative results shown.

*E. coli* ThrRS contains 13 cysteines per monomer, yet only C182 is sensitive to oxidation (Figure 2). A high-resolution crystal structure of the apo ThrRS editing domain shows that C182 is surrounded by three histidines (H73, H77 and H186), which together forms a putative metal binding site (30) (Figure 1A). The aminoacylation site of ThrRS adopts a similar architecture, with C334, H385 and H511 coordinating a structural zinc ion that is essential for amino acid selection and activation (42,43) (Figure 1B). It has been shown that purified *E. coli* ThrRS contains only one zinc per monomer (44), supporting the structural study (30) that suggests that the editing site does not tightly bind zinc. To understand the roles of histidines in C182 oxidation, we constructed and purified H73A, H77A and H186A ThrRS mutants. DAz-2 labeling experiments revealed that H73A and H186A mutations significantly decreased RSOH formation in the presence of H_2_O_2_ or NaOCl (Figure 5). In contrast, the H77A change exhibited little effect on ThrRS oxidation. Addition of ZnCl_2_ or NiCl_2_ to EDTA-treated WT ThrRS abolished C182 oxidation by H_2_O_2_, suggesting that C182 is directly activated by H73 and H186 rather than by a metal ion.

### Chronic oxidative stress leads to ThrRS mistranslation *in vivo*

**Figure 6.**
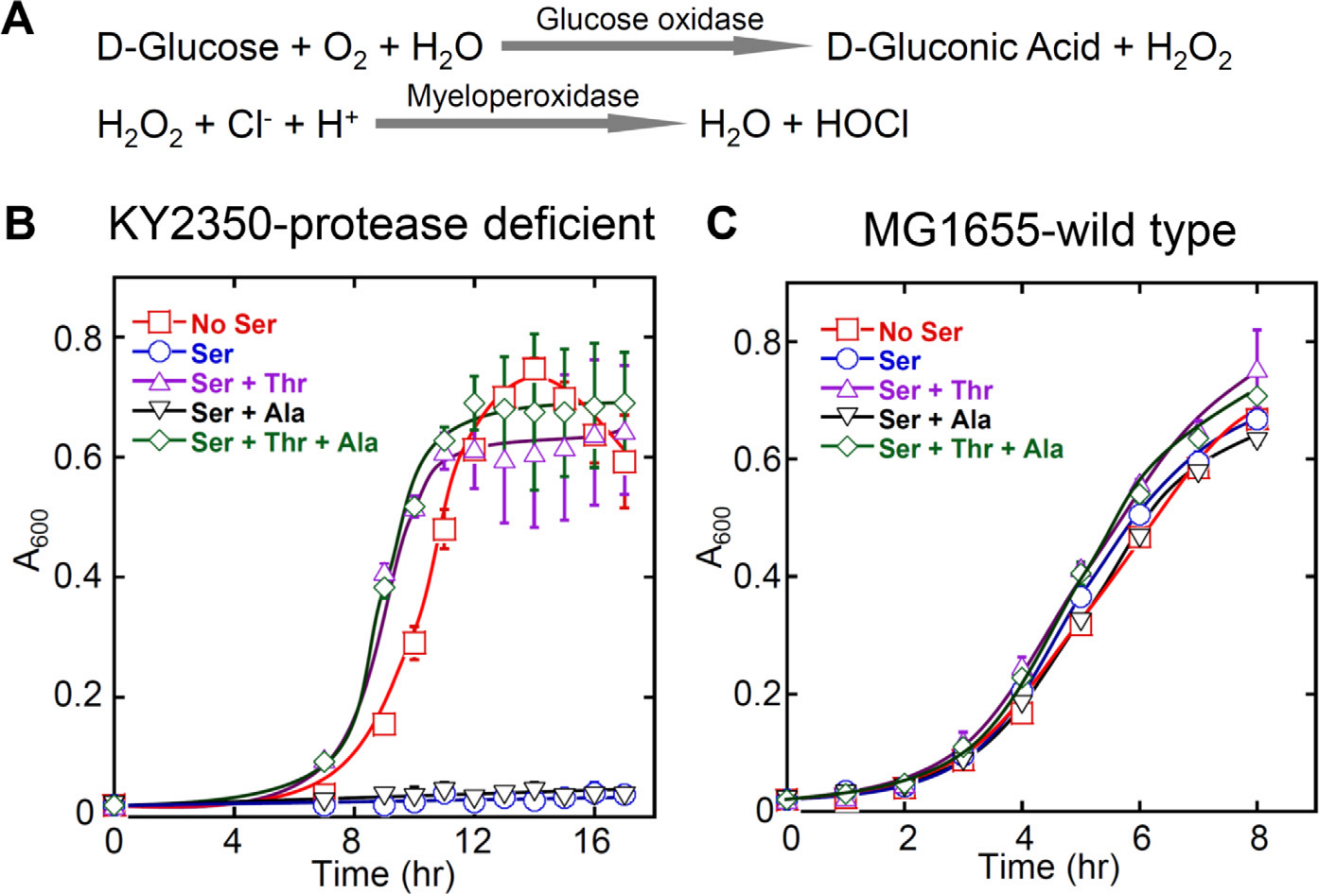
Growth of *E. coli* strains in the presence of enzyme-produced oxidants. (**A**) Scheme of H_2_O_2_ and HOCl production by glucose oxidase (0.02 U/ml) and myeloperoxidase (0.05 U/ml) in the presence of D-glucose (1%). (**B**) H_2_O_2_ and HOCl produced by glucose oxidase and myeloperoxidase inhibited the growth of KY2350 when Ser (5 mM) was added into the minimal media. Further addition of Thr (5 mM), but not Ala (5 mM), rescued the growth defect, suggesting that growth inhibition was due to increased Ser misincorporation at Thr codons. KY2350 lacks heat-shock proteases Lon, ClpP and HslVU. (**C**) WT *E. coli* MG1655 was incubated under the same conditions as KY2350, and showed increased resistance to Ser toxicity compared to KY2350. This indicates that heat-shock proteases are critical for defending against severe mistranslation caused by ThrRS editing defects. The above results were the average of at least three repeats with error bars representing standard deviations.

We have previously shown that heat-shock proteases are critical for *E. coli* to defend against mistranslation induced by a temporary dose of exogenously added H_2_O_2_ (29). The effects of chronic oxidative stress on mistranslation remain unknown. In mammals, H_2_O_2_ and hypochlorite (HOCl) are produced by oxidases and myeloperoxidase from phagocytes during host anti-microbial response (45) (Figure 6A). To mimic this natural environment and probe the impact of oxidant-induced ThrRS misacylation, we tested the growth of WT (MG1655) and protease-deficient (KY2350, which lacks the major heat-shock proteases Lon, ClpP and HslVU) *E. coli* strains in minimal media in the presence of glucose oxidase and myeloperoxidase. When H_2_O_2_ and HOCl were continuously produced, addition of Ser increased the ThrRS misacylation level and inhibited the growth of KY2350 (Figure 6B). Such levels of oxidants did not inhibit the growth of KY2350 without Ser. Further addition of Thr rescued the growth defect caused by Ser. In addition to ThrRS, AlaRS also uses a critical cysteine to edit misacylated Ser-tRNA^Ala^ (8). Supplementation of alanine (Ala) alone in the media was not able to alleviate the toxicity of Ser, indicating that Ser toxicity under oxidative stress conditions was caused by the editing defect of ThrRS but not AlaRS. In contrast to the protease-deficient strain, WT MG1655 appeared to tolerate ThrRS misacylation (Figure 6C), confirming the importance of heat-shock proteases in detoxification of mistranslated proteins.

## DISCUSSION

### Model for ThrRS oxidation

**Figure 7.**
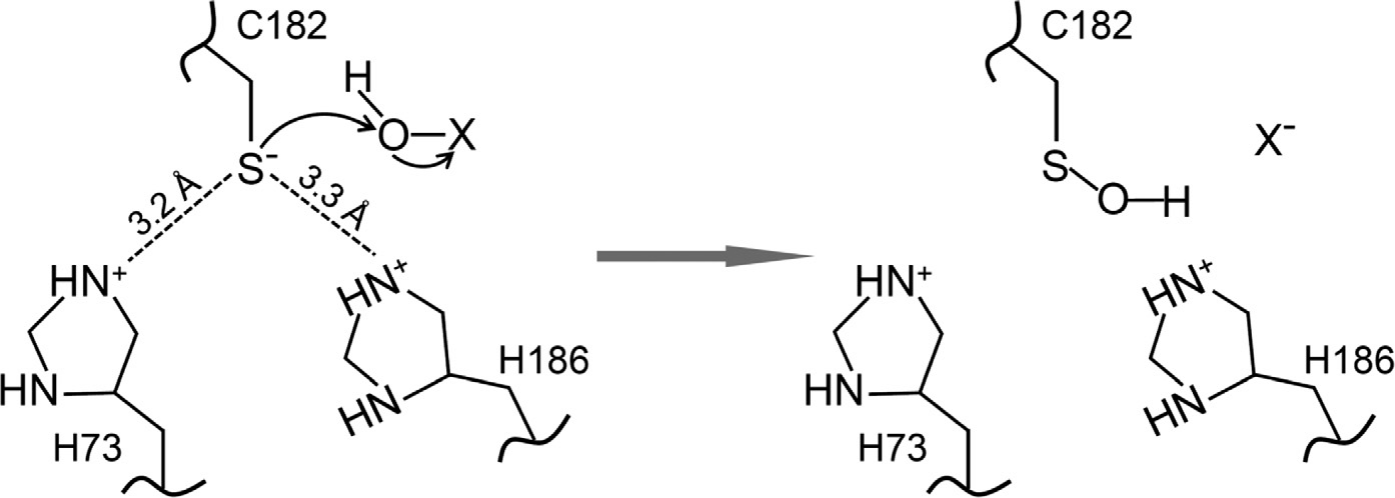
Model for ThrRS oxidation. We propose that two nitrogen atoms of H73 and H186 stabilize the deprotonated sulfur of C182, which performs nucleophilic attack to the oxidant. X stands for OH or Cl.

Protein cysteine residues are not equally sensitive to oxidation (35). Among the 13 cysteines in *E. coli* ThrRS, C182 is the only one susceptible to sulfenic acid formation. Cysteine sulfenylation requires deprotonation of the thiol group to form a thiolate, which performs nucleophilic attack to an oxidant (46). The microenvironment that promotes thiol deprotonation and lowers p*Ka* of the thiol group is thus considered favorable for cysteine activation. The crystal structure of *E. coli* ThrRS without editing substrate reveals that the sulfur atom of C182 is within hydrogen bonding distance with the imidazole rings of H73 and H186 (30) (Figure 1A). Our DAz-2 labeling experiment reveals that mutating H73 or H186 to Ala substantially decreased RSOH formation in the presence of oxidants (Figure 5). H73 and H186 are also critical for the editing activity per se, as H73A and H186A mutations decrease the *k*_cat_ value 7000- and 70-fold, respectively (31). Addition of metal ions Zn^2+^ or Ni^2+^ inhibits C182 oxidation (Figure 5), suggesting that activation of C182 is not mediated by a metal. It is therefore reasonable to propose that the side chains of H73 and H186 directly stabilize the thiolate form of C182, which attacks the oxygen of H_2_O_2_ or HOCl to form RSOH (Figure 7). It is also possible that either H73 or H186 deprotonates the thiol group. Deprotonation of C182 is supported by previous structural and kinetic studies (30,31). The crystal structure of ThrRS complexed with a post-transfer editing substrate analog shows that the sulfur of C182 interacts with the 2′-OH of A76 (30). Kinetic studies demonstrate that C182A and C182S mutations reduce the *k*_cat_ value of editing over 500-fold, strongly suggesting a catalytic role of C182 (31). Indeed, the 2′-OH of A76 is 3 Å away from a proposed catalytic water molecule (30). It is therefore likely that deprotonated C182 activates the 2′-OH to hydrolyze the editing substrate. This resembles the substrate-assisted editing mechanism of another Class II aaRS–PheRS (47). The sulfenylated form of C182 is unable to active the 2′-OH and therefore C182 oxidation results in an editing defect.

### Roles of metal ions in cysteine oxidation

Metal ions have been shown to either increase or decrease the reactivity of cysteines with oxidants (48). For instance, in Ni-containing hydrogenases, a Ni^2+^ ion is coordinated by four cysteines in the catalytic center and enhances the nucleophilic attack of the thiolate on oxidants (49). Zn^2+^ has also been suggested to enhance the nucleophilicity of a cysteine in bacterial Ada proteins (50). In contrast, Zn^2+^ inhibits cysteine oxidation in several transcription factors (48). In *E. coli* ThrRS, the aminoacylation site tightly binds a Zn ion (42), and the cysteine (C334) coordinating the Zn (Figure 1) appears to be resistant to oxidation as indicated by the lack of sulfenylation in the C182A variant (Figure 2). The editing site of ThrRS has also been suggested to loosely bind Zn, which can be removed by EDTA treatment (30). We show that EDTA treatment does not affect oxidation of C182, and addition of high concentrations of Zn^2+^ or Ni^2+^ (100 μM) inhibits RSOH formation (Figure 5). These results suggest that in *E. coli* ThrRS, metal binding protects the cysteines from oxidation.

### Oxidants leading to ThrRS misacylation

We have previously shown that H_2_O_2_ inactivates ThrRS editing and causes Ser misacylation onto tRNA^Thr^ (29). In the current work we demonstrate that ThrRS editing site is also susceptible to oxidation by air (presumably molecular oxygen) and hypochlorite (Figure 2). 1:1 molar ratio of H_2_O_2_ significantly increases C182 sulfenylation, suggesting that C182 is a sensitive redox switch. Our results *in vitro* also indicate that even air oxidation leads to increased Ser-tRNA^Thr^ production in the presence of EF-Tu, raising the intriguing possibility that *E. coli* may fine-tune translational fidelity under anaerobic and aerobic conditions. H_2_O_2_ can be generated from the respiration by-product superoxide by superoxide dismutases and is maintained at low levels during normal growth by alkyl hydroperoxidases and catalases (51). A large amount of H_2_O_2_ is released by macrophages during host-immune response, which may saturate the bacterial anti-oxidant system and cause oxidative stress (52). Hypochlorite is produced by neutrophils and is also the active ingredient of anti-microbial bleach (45,53). In bacteria, a specialized chaperone Hsp33 is used to defend against hypochlorite (53). We show that H_2_O_2_ and HOCl produced by glucose oxidase and myeloperoxidase in the media inhibit the growth of a protease-deficient strain in the presence of Ser, suggesting that Ser misincorporation at Thr codons causes protein misfolding. This pathway is distinct from protein misfolding that is directly induced by oxidative damage.

### Protein synthesis fidelity and stresses

It has been widely accepted that the flow of genetic information from DNA to protein needs to be accurate. Recent studies have revealed that protein synthesis in mycoplasma and mitochondria may be error-prone due to the absence of post-transfer editing in several aaRSs (6,54,55). It has been suggested that mistranslation may benefit mycoplasma during host-immune response by increasing the antigen diversity (54). In bacteria and yeast, methionyl-tRNA synthetase misacylates noncognate tRNAs at relatively high levels (56,57). Several stress conditions have also been shown to modulate translational fidelity. For instance, nutrient starvation increases read-through of in-frame stop codons (28); viral infection and oxidative stress enhance methionine misincorporation in mammalian cells (23). The roles of stress-induced mistranslation remain elusive. It is proposed that stress-induced translational errors may protect the cell under certain stress conditions and may therefore be considered adaptive translation (24). Lending support to this hypothesis is that ambiguous decoding increases the phenotype diversity in *Candida* (58) and enhances resistance to oxidants and heavy metals in *Saccharomyces cerevisiae* (22). Oxidant-induced ThrRS misacylation appears to be well-tolerated by WT *E. coli* (Figure 6), and ThrRS editing deficiency in *S. cerevisiae* does not cause growth defects (59). The physiological impact of stress-induced translational infidelity needs to be addressed in future studies.
